# Pattern of Mandibular Bone Invasion as a Prognostic Factor

**DOI:** 10.3390/diagnostics15232989

**Published:** 2025-11-25

**Authors:** Richard Pink, Jaroslav Michálek, Zdeněk Dvořák, Peter Tvrdý, Lenka Šašková, Michal Herman, Petr Heinz, Markéta Hermanová, Jana Zapletalová, Michal Mozoľa

**Affiliations:** 1 Department of Oral and Maxillofacial Surgery, University Hospital Olomouc, Faculty of Medicine and Dentistry, Palacky University Olomouc, 77900 Olomouc, Czech Republic; richard.pink@fnol.cz (R.P.); zdenek.dvorak@fnusa.cz (Z.D.); peter.tvrdy@fnol.cz (P.T.); lenka.saskova@fnol.cz (L.Š.); michal.herman@fnol.cz (M.H.); petr.heinz@fnol.cz (P.H.); 2 Department of Clinical and Molecular Pathology, University Hospital Olomouc, Faculty of Medicine and Dentistry, Palacky University Olomouc, 77900 Olomouc, Czech Republic; jaroslav.michalek@fnol.cz; 3 Department of Plastic and Aesthetic Surgery, St. Anne’s University Hospital, Faculty of Medicine, Masaryk University, Berkova 34, 61200 Brno, Czech Republic; 4 First Department of Pathology, Faculty of Medicine, St. Anne’s University Hospital, Faculty of Medicine, Masaryk University, 61200 Brno, Czech Republic; marketa.hermanova@fnusa.cz; 5 Department of Medical Biophysics, University Hospital, Faculty of Medicine and Dentistry, Palacky University Olomouc, 77900 Olomouc, Czech Republic; ja.zapletalova@upol.cz

**Keywords:** oral squamous cell carcinoma, mandibular bone invasion, prognosis, histopathology, survival, mandible

## Abstract

**Background/Objectives:** Mandibular bone invasion is a common and clinically relevant feature of OSCC, particularly in tumors of the lower alveolus, floor of mouth, and retromolar trigone. The prognostic value of the pattern of invasion—rather than its mere presence—remains insufficiently defined. Therefore, we evaluated the prognostic relevance of erosive vs. infiltrative mandibular invasion and the diagnostic reliability of preoperative CT. **Methods:** This retrospective, single-center observational cohort study included 83 patients with OSCC involving or adjacent to the mandible who underwent surgical resection at the Department of Oral and Maxillofacial Surgery, University Hospital Olomouc (2008–2018). Bone invasion type was classified histopathologically as erosive or infiltrative. Survival outcomes were analyzed using Kaplan–Meier and Cox regression methods. Correlation between radiologic and histologic findings was assessed using Cohen’s kappa statistics. **Results:** Mandibular invasion was confirmed in 50.6% of cases, of which roughly two-thirds were infiltrative. DSS differed across invasion groups (log-rank *p* = 0.012): infiltrative had a median DSS of 14.5 months (95% CI 0.0–32.8), no invasion had 54.2 months (CI not estimable), while erosive did not reach the median (fewer than half experienced the event). In the adjusted model (covariates: invasion type, ENE, grade, margins), infiltrative vs. no invasion was associated with worse DSS (aHR 1.93, 95% CI 1.02–3.64; *p* = 0.042); for OS, erosive vs. no invasion showed a protective association (aHR 0.39, 95% CI 0.16–0.96; *p* = 0.041). Positive/close margins were independently adverse across endpoints (e.g., DSS aHR 3.30, 95% CI 1.74–6.22). CT–histology agreement for bone invasion was κ = 0.45, indicating moderate agreement. **Conclusions:** In this retrospective single-center cohort, the pattern of mandibular bone invasion was associated with survival: infiltrative invasion aligned with worse outcomes, whereas erosive behaved similarly to no invasion, particularly for OS. Prospective, multicenter validation is warranted before routine incorporation into risk stratification or treatment selection.

## 1. Introduction

Oral squamous cell carcinoma (OSCC) is the most common malignancy of the oral cavity and remains a major global health burden, with nearly 389,000 new cases and ~188,000 deaths annually [1]. Despite advances in diagnosis and treatment, overall outcomes are suboptimal (typical 5-year survival 50–60%) and vary widely across patients [2]. Besides delayed diagnosis, biological heterogeneity plays an important role. Established etiologic factors include tobacco, alcohol, and betel quid; rising incidence in younger non-smokers suggests additional determinants such as viral, genetic, and immune influences [3]. In recognition of prognostic heterogeneity, the AJCC 8th edition incorporated depth of invasion (DOI) and extranodal extension (ENE) into oral cancer staging [4,5]. Although these refinements improved risk stratification, outcomes remain heterogeneous within the same T and N categories. Accordingly, additional histopathologic features—perineural invasion (PNI), lymphovascular invasion (LVI), tumor grade, margin status, and worst pattern of invasion—have been evaluated to further delineate risk, with inconsistent effect sizes across studies [6,7,8,9,10,11,12,13]. Bone invasion is a clinically pivotal aspect of OSCC aggressiveness, particularly for tumors of the floor of mouth, lower alveolus, and retromolar trigone. Reported mandibular invasion rates vary widely (~12–56%) depending on subsite, grade, and diagnostic criteria [14,15,16]. Under current staging, medullary (marrow) or substantial cortical invasion generally upstages to T4a, whereas superficial cortical erosion (e.g., in gingival carcinoma) is no longer automatically considered an advanced stage [4,5]. Importantly, not all bone involvement has equivalent prognostic weight. Pathologically, two principal patterns are recognized: erosive and infiltrative [9,17]. Erosive invasion shows a relatively smooth, well-demarcated front—often separated from marrow by fibrous tissue—and is thought to reflect largely osteoclast-mediated resorption. Infiltrative invasion displays irregular tongues of tumor breaching cortex and penetrating cancellous bone with minimal intervening stroma, a morphology repeatedly linked to adverse outcomes [18,19,20]. From an imaging perspective, distinguishing erosive vs. infiltrative invasion pre-operatively remains challenging. CT is essential for cortical assessment and gross medullary change, whereas MRI offers superior soft-tissue contrast but may be confounded by edema and dental artifacts [21,22,23,24]. Emerging techniques (e.g., dual-energy CT) are under evaluation, yet histopathology remains the gold standard for definitive classification [25,26]. The prognostic relevance of bone invasion has long been debated. Earlier work often treated any bone involvement as a marker of advanced disease; more recent analyses indicate that the type and extent of invasion better reflect biological behavior [18,19,20]. In particular, infiltrative/medullary invasion is consistently associated with worse disease-specific survival, whereas erosive/superficial cortical invasion may approximate outcomes of cases without bone involvement [15,16,19]. However, consensus is limited by heterogeneous definitions, mixed mandibular—maxillary cohorts, variable imaging protocols, and underuse of adjusted analyses. The mandible differs structurally and vascularly from the maxilla, complicating direct comparisons and underscoring the need for mandible-focused studies [15]. This study aimed to evaluate whether the pattern of mandibular bone invasion—erosive vs. infiltrative—is independently associated with survival in a single-center surgical OSCC cohort, while also reporting the pre-operative CT performance against histology. By focusing on a mandibular-only risk scenario and integrating established prognosticators (e.g., margins, ENE, grade, PNI/LVI, nodal burden), we sought to clarify whether the bone-invasion pattern should inform prognostic assessment and treatment planning.

## 2. Materials and Methods

### 2.1. Patient Selection

This single-center retrospective observational cohort included consecutive adult (≥18 years) patients with histologically confirmed primary oral squamous cell carcinoma (OSCC) who underwent surgical treatment at the Department of Oral and Maxillofacial Surgery, University Hospital Olomouc, between 2008 and 2018. For reporting consistency, all cases were staged (or restaged) according to the AJCC 8th edition [4].

We included tumors arising at sites with anatomical proximity to the mandible (e.g., lower alveolus/gingiva, floor of mouth, and retromolar trigone) with risk of mandibular involvement, regardless of whether bone invasion was ultimately present on histopathology. Maxillary primaries were excluded due to distinct anatomy (thin cortices, pneumatization) that affects both the pattern and detectability of bone invasion [15,23].

#### 2.1.1. Inclusion Criteria

Primary OSCC at a mandibular-adjacent site (as above); availability of preoperative head-and-neck CT, and operative and histopathology records sufficient to determine mandibular bone status.

#### 2.1.2. Exclusion Criteria

Recurrent/residual OSCC at the index site; prior head-and-neck radiotherapy or chemoradiotherapy before the index CT; prior mandibular surgery (including segmental resection) or osteoradionecrosis that could confound bone assessment; synchronous second primary head-and-neck cancer; Non-OSCC histology; distant metastasis when treated non-curatively; insufficient documentation for key variables.

All patients underwent preoperative CT before surgery. Histopathology served as the reference standard for mandibular bone status and subsequent classification of invasion pattern (detailed in Section 2.2). All patients underwent surgical resection of the primary tumor; the extent of mandibular resection (marginal vs. segmental) reflected preoperative imaging and intraoperative findings. Adjuvant therapy (postoperative RT or concurrent CRT) was indicated by a multidisciplinary tumor board in accordance with contemporaneous national guidance (Czech Oncology Society ‘Modrá kniha’; earlier editions published as ‘Zásady cytostatické léčby maligních onkologických onemocnění’) and international guidance (NCCN Head and Neck), applicable to the 2008–2018 treatment period.

### 2.2. Histopathological Examination Protocol

**Figure 1:**
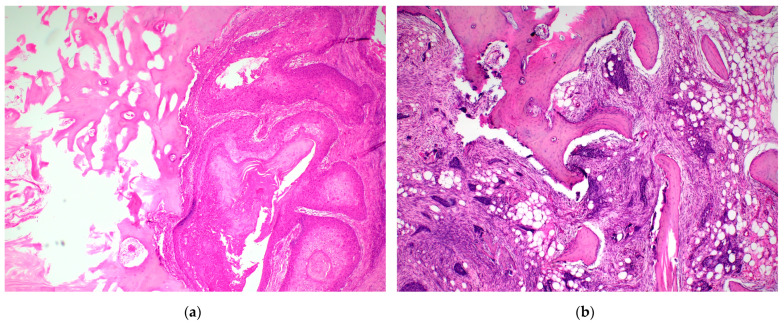
Histopathological patterns of mandibular bone invasion in oral squamous cell carcinoma. (**a**) Erosive invasion—Tumor front shows a smooth, pushing interface with the mandibular cortex, separated from the underlying cancellous bone by a fibrous connective tissue layer. There is no penetration of tumor islands into medullary spaces. This pattern corresponds to a less aggressive growth mode. (**b**) Infiltrative invasion—Irregular, finger-like cords and nests of squamous carcinoma penetrate deeply into the cancellous bone, often accompanied by bone resorption and destruction of trabecular structure. This pattern reflects a more aggressive and destructive biological behavior.

All surgical mandibular resection specimens were immediately sent to the Institute of Clinical and Molecular Pathology, University Hospital Olomouc, for processing according to a standardized protocol [27]. Each specimen was fixed in 10% neutral buffered formalin for at least 24 h. Prior to fixation, the specimen orientation was marked collaboratively by the surgeon and pathologist. Resection margins were inked separately for soft tissues (mucosa, skin) and bone at osteotomy sites. Bone samples were decalcified in 10% formic acid until adequately softened. Complete decalcification is essential for accurate microscopic evaluation of tumor–bone interfaces and resection margins [15]. Because the process requires several days to weeks, intraoperative frozen section assessment of bone margins was not routinely feasible. Following decalcification, tissues were embedded in paraffin and sectioned at 4–5 µm. Sections were stained with hematoxylin–eosin (H&E). Additional immunohistochemical staining (e.g., cytokeratin) was performed as needed to confirm tumor invasion. Information regarding the presence and type of mandibular bone invasion was conducted by a single expert pathologist who performed the retrospective slide review. Two types of invasion were defined according to established histopathologic criteria [15,17,28] (Figure 1).

Erosive (superficial) invasion: tumor eroding the cortical surface, separated from the medullary cavity by fibrous tissue, without deep penetration into cancellous bone.

Infiltrative (deep) invasion: tumor penetrating the full cortical thickness with irregular invasion into cancellous bone, lacking an intervening fibrous layer.

Classification was performed through blinded histopathological review by an experienced pathologist unaware of clinical outcomes [JM]. In equivocal cases (e.g., fragmented samples or inflammatory changes), an additional pathologist was consulted, and consensus was reached. Because classification was performed by a single expert pathologist, with a second pathologist consulted only in equivocal cases, a formal interobserver κ could not be calculated. We acknowledge this limitation and have specified our consensus procedure for transparency.

### 2.3. Statistical Analysis

All analyses were performed in IBM SPSS Statistics v23.0 (IBM Corp., Armonk, NY, USA). Two-sided *p* < 0.05 was considered statistically significant. Endpoints were defined as follows: disease-specific survival (DSS)—time from surgery to death from OSCC; disease-free survival (DFS)—time to first locoregional recurrence/progression; and overall survival (OS)—time to death from any cause. Analyses were conducted on available cases (no imputation).

Survival analysis—Time-to-event distributions were estimated by Kaplan–Meier. Group differences across three bone invasion categories (none, erosive, and infiltrative) were tested with the log-rank test. When the overall test was significant, pairwise log-rank comparisons were performed with Holm’s adjustment for multiple testing. We report medians with 95% CIs (and 5-year survival where estimable). Where the median could not be estimated, this is stated explicitly.

Multivariable analysis—We fitted Cox proportional hazards (PH) models with the ENTER entry for DSS (primary) and DFS/OS (secondary). Prespecified covariates were as follows: type of invasion (reference = no invasion; indicator terms for erosive and infiltrative), extranodal extension (ENE) (present vs. absent), tumor grade (modelled as G3 vs. G1–2), and surgical margins (positive/close vs. negative). The composite AJCC Stage (I–IV) was examined, but violated the PH assumption; therefore, the primary adjusted model excluded stage [4]. We did not use stepwise selection given the sample size (*n* = 83). PH assumptions were evaluated by log–minus–log plots and time-dependent covariate checks in SPSS; no material violations were seen among retained covariates.

Radiologic–pathologic agreement—Pre-operative CT was analyzed binary for presence/absence of mandibular invasion; histopathology served as the reference standard. Agreement was summarized by Cohen’s κ with 95% CI, overall percentage agreement (95% CI), and McNemar’s test for asymmetry [29]. For clinical interpretability, we additionally report sensitivity, specificity (95% CIs). Pattern-level (erosive vs. infiltrative) calls were not attempted radiologically.

Because the mixed pattern commonly contains medullary/infiltrative foci and shows outcomes intermediate but trending toward those of infiltrative invasion, we a priori pooled mixed with infiltrative for survival analyses. This classifies tumors by the most aggressive component at the tumor–bone interface, minimizing the risk of misclassification.

## 3. Results

### 3.1. Patient and Tumor Characteristics

The study cohort included 83 patients (62 men, 21 women) with a median age of 61 years (range 31–84 years). The most frequent primary tumor sites were the floor of the mouth with mandibular involvement (34.9%), lower gingiva (30.1%), and retromolar region (16.9%). All tumors were histologically confirmed squamous cell carcinomas. Histological grading revealed 20.5% well differentiated, 36.1% moderately differentiated, and 43.4% poorly differentiated (grade 3) tumors. Bone invasion was histopathologically verified in 42 patients (50.6%). Among them, 28 (66.7%) exhibited the infiltrative type and 14 (33.3%) the erosive type of mandibular invasion. Ten cases showing mixed characteristics were classified as infiltrative for statistical purposes. Regarding surgical management, segmental mandibulectomy was performed in 69.9% of cases, while marginal mandibulectomy was used in the remaining patients. Adjuvant therapy included radiotherapy alone in 27 patients (32.5%) and concurrent chemoradiotherapy in 33 patients (39.8%).

### 3.2. Survival Outcomes

The median follow-up period was 90 months. During this time, 46 patients (55.4%) died of disease, 54 (65%) developed locoregional recurrence or progression, and 20 (24%) presented with distant metastases. For the entire cohort, median DSS was 42.3 months (95% CI 18.7–65.8), median DFS 15.4 months (0.0–35.7), and median OS 29.0 months (12.8–45.2). Where the median was estimable, we report it with 95% CI; means are shown only as ancillary summaries (Table 1).

**Figure 2:**
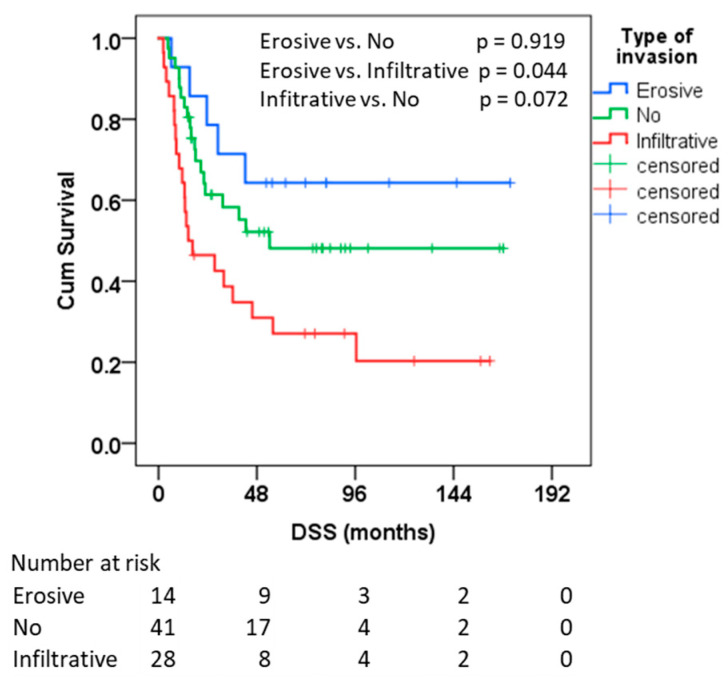
Kaplan-Meier analysis for DSS for the type of bone invasion in months. Numbers at risk for each group are shown beneath the *x*-axis.

The Kaplan–Meier analysis (Figure 2, Table 2) demonstrated significant differences in disease-specific survival (DSS) among the three groups defined by bone invasion type.

For DSS, the infiltrative group had a median of 14.5 months (95% CI 0.0–32.8); the erosive group’s median was not reached (fewer than half experienced the event by the end of the follow-up, with a mean of 118.6 months), and the no-invasion group showed a median of 54.2 months (CI not estimable). For OS, medians were 14.5 months (8.8–20.1) for infiltrative, 26.1 months (4.7–47.5) for no-invasion, and not reached for erosive. Group differences were significant for DSS overall, and post hoc testing showed longer OS for erosive vs. infiltrative. We do not use means except where the median is not estimable (i.e., the Kaplan–Meier curve does not drop below 0.5); in such instances, the mean is reported only for context.

Across all endpoints, positive margins were the strongest independent adverse factor. Grade G3 (vs. G1–2) remained independently adverse for DSS and OS and was borderline for DFS. ENE did not retain independent significance after adjustment. As a factor, invasion type was associated with DSS and OS but not DFS: infiltrative invasion was independently associated with worse DSS versus no invasion, whereas erosive invasion showed a protective association for OS versus no invasion. Full adjusted HRs and 95% CIs are reported in Table 3; the results were directionally robust in sensitivity analyses.

### 3.3. Radiologic–Pathologic Correlation

Comparison between CT findings and histopathological confirmation of mandibular invasion showed moderate agreement, with a Cohen’s κ = 0.449 (95% CI 0.250–0.649) and an overall concordance of 72.7% (95% CI 61.9–81.4%).

McNemar’s test indicated no significant systematic bias between CT and histological assessment (*p* = 0.664) (Table 4).

## 4. Discussion

In this single-center cohort of oral squamous cell carcinoma (OSCC) at risk for mandibular involvement, the pattern of bone invasion was associated with outcome. On Kaplan–Meier analysis, patients with infiltrative invasion experienced inferior disease-specific survival (DSS) compared with those with erosive invasion or no invasion; in multivariable Cox models that adjusted for margins, grade, and ENE, infiltrative vs. no invasion remained adverse for DSS. We caution that the erosive subgroup was small (*n* = 14), which widens confidence intervals and limits precision. For example, the OS association for erosive vs. no invasion (aHR 0.394, 95% CI 0.161–0.962) suggests a protective effect but should be interpreted cautiously given the sample size; the DSS estimate (aHR 0.520, 95% CI 0.191–1.421) was not statistically significant. These data are therefore hypothesis-generating and require confirmation in larger cohorts. Across endpoints, positive/close margins and high histological grade (G3) were the most consistent independent predictors. These findings indicate that the prognostic signal is not conveyed by the presence of bone invasion alone, but by its morphology and the context of co-existing risk factors [15,17,18,19].

The distinction between erosive and infiltrative invasion reflects different tumor–bone interactions observed histologically: erosive fronts tend to be smoother, often separated from marrow by fibrous tissue and compatible with a predominantly osteoclast-mediated resorptive process; infiltrative fronts show irregular epithelial tongues and nests breaching cortex and extending into cancellous bone with minimal intervening stroma—features that correlate with more aggressive local behavior. While molecular correlates (e.g., EMT-like shifts, MMP activity, and altered adhesion) have been described, we emphasize here the clinically actionable morphology rather than extended mechanistic detail [20,22].

Relation to contemporary literature. Our data align with reports that move beyond a binary definition of “bone invasion.” Ebrahimi et al. highlighted prognostic and staging implications of depth/extent of osseous involvement [14]. More recently, Mahajan et al. proposed marrow and mandibular canal-oriented descriptors and showed that medullary involvement associates with worse survival—consistent with our DSS results [30]. The Li et al. meta analysis similarly concluded that deeper/medullary patterns carry a higher risk than superficial cortical erosion [19]. Our mandible-focused cohort supports these conclusions and adds adjusted estimates as well as sensitivity analyses demonstrating that mixed cases behave like infiltrative disease; excluding or reclassifying them did not alter the inference.

Imaging–pathology agreement and clinical reading. Preoperative CT showed moderate agreement with histopathology (κ ≈ 0.45), which is in line with mixed accuracies reported for CT/MRI in mandibular invasion [21,22,23,24]. In our protocol, CT was analyzed binary (any invasion vs. none); pattern-level calls were not attempted radiologically. Sources of discordance include subtle early marrow change, dental artifacts, and post-inflammatory signal. While dual energy CT and quantitative MRI offer promise for better preoperative discrimination, histopathology remains the reference standard [25,26]. Accordingly, preoperative imaging should be interpreted through multidisciplinary review, where the suspected pattern (erosive vs. infiltrative) would change the planned extent of bone resection [26].

Implications for surgical strategy (explicitly linked to our data). In our adjusted models, margins and grade dominated prognosis, and the invasion pattern provided incremental prognostic information. Practically, isolated erosive involvement—especially when clear bony and soft tissue margins are achievable and no additional high-risk features (e.g., ENE, PNI/LVI, and multiple nodes) are present—may be appropriate for marginal mandibulectomy, preserving mandibular continuity without evident compromise of oncologic control. In contrast, infiltrative invasion, which in our cohort aligned with inferior DSS, supports segmental resection when feasible and a lower threshold for adjuvant therapy if other risk features coexist. These recommendations are consistent with prior series [15,16,18,19,20] and with literature on margin adequacy and margin-to-DOI metrics [31,32,33,34,35]. Here are anchored to our survival medians and adjusted effects. Notably, the AJCC 8th decision to exclude superficial cortical erosion from automatic T4a is concordant with the observed outcome proximity between erosive and no invasion in our series.

Integration with established prognosticators. Our results complement classic risk markers—margin status, grade, nodal burden/ENE, PNI/LVI [8,9,10,11,12,13,31,32,33,34,35,36,37,38,39,40,41,42]—which remain the backbone of risk stratification. The invasion pattern can be considered alongside depth of invasion, quantitative nodal indices, and margin to depth ratios to refine prognostic models [7,43]. Conceptually, it parallels the “worst pattern of invasion” at the soft tissue front: both variables capture intrinsic invasiveness that may not be fully reflected by T/N categories alone [44].

Strengths and limitations. Strengths include a mandible-specific cohort, standardized histopathologic definitions of bone invasion, multivariable survival modeling with a priori covariates, and prespecified handling of mixed cases with robustness checks (KM and adjusted analyses). Limitations are the retrospective, single-center design; absence of centralized/consensus imaging review (single radiology reader; no interobserver κ), and potential temporal heterogeneity in imaging protocols across 2008–2018. We could not estimate interobserver κ for histopathological patterning because classification was performed by a single reader, with consensus only in equivocal cases. A small erosive subgroup, which widens CIs and limits power for secondary endpoints (e.g., DFS). Proportional hazards assumptions limited the role of the composite stage in the primary model. Residual confounding cannot be excluded.

Future directions. Prospective, multicenter studies with standardized CT/MRI acquisition and blinded multi-reader imaging, together with interobserver pathology assessment, are needed to validate these findings. Quantitative descriptors (e.g., depth/volume of medullary replacement, proportion of cancellous bone replaced) and radiomics/AI may improve preoperative prediction of pattern. Finally, linking invasion pattern to function and quality of life after marginal vs. segmental resections would help balance oncologic safety against morbidity in everyday decision making. In summary, in this retrospective cohort, the pattern of mandibular bone invasion—considered together with margins and grade—was associated with survival and may help tailor both the extent of bony resection and adjuvant therapy. Given sample size and design constraints, these estimates should be interpreted cautiously and validated in prospective multicenter cohorts.

## 5. Conclusions

In this single-center retrospective cohort, the histopathologic pattern of mandibular bone invasion was associated with outcomes: after adjustment for clinicopathologic covariates, infiltrative invasion was linked to worse disease-specific survival, whereas erosive invasion behaved more similarly to no bone involvement and was not independently associated with adverse outcomes. These findings support reporting the invasion pattern in routine histopathology and may inform multidisciplinary decisions regarding surgical approach and adjuvant therapy; however, they should be interpreted cautiously given the study design and the small number of erosive cases.

Prospective, adequately powered, multicenter studies with standardized imaging and pathology protocols are required to validate and quantify this prognostic effect and to refine risk-stratification models in mandibular OSCC. Bone-invasion type may represent a clinically relevant prognostic factor.

## Figures and Tables

**Table 1:** Cohort-level survival times (Kaplan–Meier).

	Mean	95% CI for Mean	Median	95% CI for Median
DSS (months)	83.0	65.8–100.3	42.3	18.7–65.8
DFS (months)	66.8	50.0–83.5	15.4	0.0–35.7
OS (months)	62.7	48.5–77.0	29.0	12.8–45.2

**Table 2:** Kaplan–Meier DSS by mandibular invasion pattern. Median is not estimable in the erosive invasion group (KM curve does not fall below 0.5 within follow-up). DSS (disease-specific survival): event = death related to the diagnosis; DFS (disease-free survival): event = recurrence; OS (overall survival): event = death from any cause.

DSS (Months)	Mean	95% CI for Mean	Median	95% CI for Median
No invasion	92.2	67.7–116.6	54.2	-
Infiltrative invasion	51.9	28.7–75.1	14.5	0.0–32.8
Erosive invasion	118.6	81.2–156.1	-	

**Table 3:** Multivariable Cox proportional hazards models (ENTER): adjusted HRs for DSS, DFS, and OS, Type of invasion 0 = no invasion; Abbreviations: DSS—disease-specific survival; DFS—disease-free survival; OS—overall survival; HR—hazard ratio; CI—confidence interval. ENE—Extra Nodal Extension.

	DSS			DFS			OS		
	HR	95% Pro HR	*p*-Value	HR	95% Pro HR	*p*-Value	HR	95% Pro HR	*p*-Value
Type of invasion 0	1		**0.017**	1		0.163	1		**0.021**
infiltrative	**1.930**	1.023–3.640	**0.042**	1.577	0.871–2.855	0.133	1.416	0.822–2.436	0.210
erosive	0.520	0.191–1.421	0.202	0.786	0.345–1.790	0.567	**0.394**	0.161–0.962	**0.041**
Positive surgical margin	**3.295**	1.744–6.224	**0.0002**	**2.541**	1.441–4.479	**0.001**	**2.484**	1.471–4.195	**0.001**
Grade G3 (vs. Grade 1–2)	**2.081**	1.123–3.856	**0.020**	1.715	0.978–3.006	0.060	**1.742**	1.031–2.941	**0.038**
ENE (present)	1.681	0.908–3.113	0.098	1.281	0.716–2.292	0.404	1.561	0.909–2.680	0.106

**Table 4:** Agreement between preoperative CT assessment and histopathological confirmation of mandibular bone invasion.

	Cohen’s Kappa (95% CI) Classification	% of Agreement (95% CI)	McNemar’s Test	Sensitivity	Specificity
CT invasion vs. Type of invasion (0, infitrative+erosive)	0.449 (0.250–0.649) moderate agreement	72.7% (61.9–81.4%)	0.664	78.57%	68.29%

## Data Availability

The data presented in this study are available on request from the corresponding author. The data are not publicly available due to privacy and ethical restrictions.

## References

[1] Sung H., Ferlay J., Siegel R.L., Laversanne M., Soerjomataram I., Jemal A., Bray F. (2021). Global Cancer Statistics 2020: GLOBOCAN estimates of incidence and mortality worldwide for 36 cancers in 185 countries. CA Cancer J. Clin..

[2] Rivera C. (2015). Essentials of oral cancer. Int. J. Clin. Exp. Pathol..

[3] Warnakulasuriya S. (2009). Causes of oral cancer—An appraisal of controversies. Br. Dent. J..

[4] Amin M.B., Edge S.B., Greene F.L., Byrd D.R., Brookland R.K., Washington M.K., Gershenwald J.E., Compton C.C., Hess K.R., Sullivan D.C. (2017). AJCC Cancer Staging Manual.

[5] Zanoni D.K., Patel S.G., Shah J.P. (2019). Changes in the 8th Edition of the American Joint Committee on Cancer (AJCC) staging of head and neck cancer: Rationale and implications. Curr. Oncol. Rep..

[6] Almangush A., Bello I.O., Keski–Säntti H., Mäkinen L.K., Kauppila J.H., Pukkila M., Hagström J., Laranne J., Tommola S., Nieminen O. (2014). Depth of invasion, tumor budding, and worst pattern of invasion: Prognostic indicators in early-stage oral tongue cancer. Head Neck.

[7] Almangush A., Pirinen M., Heikkinen I., A Mäkitie A., Salo T., Leivo I. (2018). Tumour budding in oral squamous cell carcinoma: A meta-analysis. Br. J. Cancer.

[8] Binmadi N., Alsharif M., Almazrooa S., Aljohani S., Akeel S., Osailan S., Shahzad M., Elias W., Mair Y. (2023). Perineural invasion is a significant prognostic factor in OSCC: Systematic review/meta-analysis. Diagnostics.

[9] Huang S., Zhu Y., Cai H., Zhang Y., Hou J. (2021). Impact of lymphovascular invasion in OSCC: Meta-analysis. Oral Surg. Oral Med. Oral Pathol. Oral Radiol..

[10] Caponio V.C.A., Troiano G., Togni L., Zhurakivska K., Santarelli A., Laino L., Rubini C., Muzio L.L., Mascitti M. (2023). Pattern/localization of perineural invasion predicts poor survival in oral tongue carcinoma. Oral Dis..

[11] Chen T.-C., Wang C.-P., Ko J.-Y., Yang T.-L., Hsu C.-W., Yeh K.-A., Chang Y.-L., Lou P.-J. (2013). Impact of PNI and/or LVI on survival of early-stage OSCC. Ann. Surg. Oncol..

[12] Quintana D.M.V.O., Dedivitis R.A., Kowalski L.P. (2022). Prognostic impact of perineural invasion in oral cancer: Systematic review. Acta Otorhinolaryngol. Ital..

[13] Li J., Liu S., Li Z., Han X., Que L. (2021). Perineural invasion and its role in survival stratification of OSCC patients. Front. Oncol..

[14] Ebrahimi A., Murali R., Gao K., Elliott M.S., Clark J.R. (2011). The prognostic and staging implications of bone invasion in oral squamous cell carcinoma. Cancer.

[15] Brown J.S., Lowe D., Kalavrezos N., D’Souza J., Magennis P., Woolgar J. (2002). Patterns of invasion and routes of tumor entry into the mandible by oral squamous cell carcinoma. Head Neck.

[16] Ash C.S., Nason R.W., Abdoh A.A., Cohen M.A. (2000). Prognostic implications of mandibular invasion in oral cancer. Head Neck.

[17] Wong R.J., Keel S.B., Glynn R.J., Varvares M.A. (2000). Histological pattern of mandibular invasion by oral squamous cell carcinoma. Laryngoscope.

[18] Okura M., Yanamoto S., Umeda M., Otsuru M., Ota Y., Kurita H., Kamata T., Kirita T., Yamakawa N., Yamashita T. (2016). Prognostic and staging implications of mandibular canal invasion in lower gingival squamous cell carcinoma. Cancer Med..

[19] Li C., Lin J., Men Y., Yang W., Mi F., Li L. (2017). Does Medullary Versus Cortical Invasion of the Mandible Affect Prognosis in Patients With Oral Squamous Cell Carcinoma?. J. Oral Maxillofac. Surg..

[20] Vaassen L.A., Speel E.-J.M., Kessler P.A.W.H. (2017). Bone invasion by oral squamous cell carcinoma: Molecular alterations leading to osteoclastogenesis—A review literature. J. Cranio-Maxillofac. Surg..

[21] Vidiri A., Guerrisi A., Pellini R., Manciocco V., Covello R., Mattioni O., Guerrisi I., Di Giovanni S., Spriano G., Crecco M. (2010). MDCT and MRI in the evaluation of mandibular invasion by oral cavity SCC: Correlation with pathology. J. Exp. Clin. Cancer Res..

[22] Imaizumi A., Yoshino N., Yamada I., Nagumo K., Amagasa T., Omura K., Okada N., Kurabayashi T. (2006). A potential pitfall of MR imaging for assessing mandibular invasion of squamous cell carcinoma in the oral cavity. AJNR Am. J. Neuroradiol..

[23] Nae A., O’LEary G., Feeley L., Fives C., Fitzgerald B., Chiriac E., Sheahan P. (2019). Utility of CT and MRI in assessment of mandibular involvement in oral cavity cancer. World J. Otorhinolaryngol. Head. Neck Surg..

[24] Gu D.H., Yoon D.Y., Park C.H., Chang S.K., Lim K.J., Seo Y.L., Yun E.J., Choi C.S., Bae S.H. (2010). CT, MR, 18F-FDG PET/CT, and their combined use for assessment of mandibular invasion by oral cavity SCC. Acta Radiol..

[25] Timmer V.C.M.L., Crombag G.A.J.C., van Kuijk S.M.J., Vaassen L.A.A., Kessler P.A.W.H., Postma A.A. (2025). The accuracy of dual energy CT on evaluation of bone invasion caused by oral squamous cell carcinoma—A comparison to MRI. J. Craniomaxillofac. Surg..

[26] Jo G.-D., Oh K.-Y., Kim J.-E., Yi W.-J., Heo M.-S., Lee S.-S., Huh K.-H. (2024). Underlying bone change in oral squamous cell carcinoma observed from MRI and CT: Implications for aggressiveness and prognosis. J Dent Sci..

[27] College of American Pathologists (CAP) Protocol for the Examination of Specimens from Patients with Cancers of Oral Cavity. June 2023. https://www.cap.org/protocols-and-guidelines/cancer-reporting-tools/cancer-protocol-templates.

[28] Slootweg P.J., Müller H. (1989). Mandibular invasion by oral squamous cell carcinoma. J. Cranio-Maxillofac. Surg..

[29] McHugh M.L. (2012). Interrater reliability: The kappa statistic. Biochem. Med..

[30] Mahajan A., Dhone N., Vaish R., Singhania A., Malik A., Prabhash K., Ahuja A., Sable N., Chaturvedi P., Noronha V. (2022). Prognostic impact of pattern of mandibular involvement in GBC-SCC: Marrow/mandibular-canal staging. Front. Oncol..

[31] Kang C.-J., Wen Y.-W., Lee S.-R., Lee L.-Y., Hsueh C., Lin C.-Y., Fan K.-H., Wang H.-M., Hsieh C.-H., Ng S.-H. (2021). Surgical margins status and prognosis after resection of oral cavity SCC: Nationwide registry study. Cancers.

[32] Hung C.-Y., Lee T.-L., Chang C.-W., Wang C.-P., Lin M.-C., Lou P.-J., Chen T.-C. (2024). Margin to depth-of-invasion ratio as an indicator for stratifying close margins in early-stage OSCC. Oral Oncol..

[33] Sultania M., Chaudhary I., Jain P., Ghalige H., Rajan D., Sudhakar G., Raghuram K., Muduly D., Barik S., Pathak M. (2023). Margin to Depth of Invasion Ratio: Predictor of survival in oral cancer. JCO Glob. Oncol..

[34] Liu T., Clark J., David M., David M., Schache A., Bajwa M.S., Low T.H., Gupta R., Batstone M.D. (2025). The Impact of Stratified Surgical Margins on Survival Outcomes in Oral Cavity Squamous Cell Carcinoma: A Multicenter Analysis. Head Neck.

[35] Klibngern H., Kang C.-J., Lee L.-Y., Ng S.-H., Lin C.-Y., Fan K.-H., Chen W.-C., Lin J.-C., Tsai Y.-T., Lee S.-R. (2024). Margin-to-depth ratio as an independent prognostic factor in resected OSCC: Nationwide cohort. Oral Oncol..

[36] Liao L.-J., Lu C.-L., Cheng Y.-P., Cheng P.-C., Chen Y.-C., Chiang C.-J., Lee W.-C., You S.-L., Hsu W.-L. (2025). Cervical lymph-node positive probability predicts survival in OSCC: Nationwide study. Cancers.

[37] Tsai T., Iandelli A., Marchi F., Huang Y., Tai S., Hung S., Kao H., Chang K. (2022). Prognostic value of lymph-node burden in oral cavity cancer: Meta-analysis. Laryngoscope.

[38] Huang T.H., Li K.Y., Choi W.S. (2019). Lymph node ratio as a prognostic variable in OSCC: Meta-analysis. Oral Oncol..

[39] Mermod M., Tolstonog G., Simon C., Monnier Y. (2016). Extracapsular spread in HNSCC: Systematic review and meta-analysis. Oral Oncol..

[40] Henson C.E., Abou-Foul A.K., Morton D.J., McDowell L., Baliga S., Bates J., Lee A., Bonomo P., Szturz P., Nankivell P. (2023). Extranodal extension in HNC: State-of-the-art review and gap analysis. Front. Oncol..

[41] Shaw R.J., Lowe D., Woolgar J.A., Brown J.S., Vaughan E.D., Evans C., Lewis-Jones H., Hanlon R., Hall G.L., Rogers S.N. (2010). Extracapsular spread in OSCC. Head Neck.

[42] Ferlito A., Rinaldo A., Devaney K.O., MacLennan K., Myers J.N., Petruzzelli G.J., Shaha A.R., Genden E.M., Johnson J.T., de Carvalho M.B. (2002). Prognostic significance of microscopic/macroscopic extracapsular spread in cervical nodes. Oral Oncol..

[43] Mascitti M., Togni L., Caponio V.C.A., Zhurakivska K., Muzio L.L., Rubini C., Santarelli A., Troiano G. (2023). Prognostic significance of tumor budding thresholds in oral tongue SCC. Oral Dis..

[44] Naegeli-Pullankavumkal C., Ferrari R., Gander T., Lanzer M. (2025). Staging and treatment implications in small oral SCC with bone infiltration. Biomedicines.

